# Age-related sensitive periods influence visual language discrimination in adults

**DOI:** 10.3389/fnsys.2013.00086

**Published:** 2013-11-13

**Authors:** Whitney M. Weikum, Athena Vouloumanos, Jordi Navarra, Salvador Soto-Faraco, Núria Sebastián-Gallés, Janet F. Werker

**Affiliations:** ^1^Department of Pediatrics, University of British ColumbiaVancouver, BC, Canada; ^2^Department of Psychology, New York UniversityNew York, NY, USA; ^3^Parc Sanitari Sant Joan de Déu, CIBERSAM, Fundació Sant Joan de DéuBarcelona, Spain; ^4^Center for Brain and Cognition, Departament de Tecnologies de la Informació i les Comunicacions, Universitat Pompeu FabraBarcelona, Spain; ^5^Institució Catalana de Recerca i Estudis AvançatsBarcelona, Spain; ^6^Department of Psychology, University of British ColumbiaVancouver, BC, Canada

**Keywords:** visual speech, language discrimination, sensitive period, adults, age of acquisition

## Abstract

Adults as well as infants have the capacity to discriminate languages based on visual speech alone. Here, we investigated whether adults' ability to discriminate languages based on visual speech cues is influenced by the age of language acquisition. Adult participants who had all learned English (as a first or second language) but did not speak French were shown faces of bilingual (French/English) speakers silently reciting sentences in either language. Using only visual speech information, adults who had learned English from birth or as a second language before the age of 6 could discriminate between French and English significantly better than chance. However, adults who had learned English as a second language after age 6 failed to discriminate these two languages, suggesting that early childhood exposure is crucial for using relevant visual speech information to separate languages visually. These findings raise the possibility that lowered sensitivity to non-native visual speech cues may contribute to the difficulties encountered when learning a new language in adulthood.

## Introduction

From the first days of life, language perception involves both auditory and visual speech information. The visual information available in talking faces contains linguistic cues often correlated with and complementary to the acoustic signal (e.g., Munhall and Vatikiotis-Bateson, 1998; Yehia et al., 1998). In adults, seeing talking faces enhances speech perception (Sumby and Pollack, 1954), and in some cases, can perceptually dominate overheard speech (see McGurk and MacDonald, 1976; Campbell, 2009). Similarly, there is evidence suggesting that very young infants can match heard speech with the corresponding talking faces (Kuhl and Meltzoff, 1982; Patterson and Werker, 2002), detect a mismatch between heard and seen speech (Kushnerenko et al., 2008; Bristow et al., 2009), and integrate mismatching audiovisual speech (Rosenblum et al., 1997; Burnham and Dodd, 2004; Desjardins and Werker, 2004). Moreover, both adults and young infants are able to discriminate between languages just from silent talking faces (Soto-Faraco et al., 2007; Weikum et al., 2007; Ronquest et al., 2010).

Sensitive periods in language development have been documented for both auditory and visual speech perception. Infants begin life with broad perceptual sensitivities that support learning phonetic properties from many of the world's languages (e.g., Saffran et al., 2006), but as their experience accumulates across the first year of life, their perceptual sensitivities become attuned to match the language(s) present in their environment (see Werker and Tees, 2005, for a review). This pattern is seen in age-related changes between 6 and 10 months of age for the discrimination of minimal pairs that are phonologically relevant to the infant's native language (e.g., Werker and Tees, 1984; Werker and Lalonde, 1988; Best et al., 1995; Bosch and Sebastián-Gallés, 2003; Tsao et al., 2006; Albareda-Castellot et al., 2011), in visual language discrimination (Weikum et al., 2007; Sebastián-Gallés et al., 2012), and even in auditory-visual matching (Pons et al., 2009). This tendency, often referred to as “perceptual narrowing” (Scott et al., 2007), seems to be extensively constrained by maturational factors, particularly in the domain of phonetic consonant discrimination (Peña et al., 2012).

An interesting case is when the listener is regularly exposed to more than one language (as is arguably the case for most of the world's population; see Brutt-Griffler and Varghese, 2004). Infants exposed to two different languages seem to maintain their sensitivity to the distinctions used in each of their languages. For example, at the end of the first year of life, bilingual infants can discriminate the heard speech sounds (Bosch and Sebastián-Gallés, 2003; Burns et al., 2003; Albareda-Castellot et al., 2011) and visual speech (Weikum et al., 2007) of both of their native languages. Thus, early life exposure to two languages results in a perceptual system that reflects, and is responsive to, the input from each language.

In stark contrast to the flexibility that “crib” bilinguals show, individuals who acquire a second language in adulthood have notorious difficulty learning to discriminate some of the phonological categories in their second language (L2). One of the best known examples is the difficulty Japanese learners often have in discriminating the English /r/ vs. /l/ contrast (Goto, 1971). It is equally hard for English speakers to learn to discriminate the dental /da/ vs. retroflex /Da/ sounds used in Hindi (Werker et al., 1981). In both cases, while intensive training can lead to some improvement, performance does not reach the level of native speakers (Tees and Werker, 1984; Lively et al., 1993; McClelland et al., 2002). Even highly proficient bilinguals, such as Spanish-native speakers of Catalan, can learn to discriminate contrasts specific to their L2 (i.e., /e/ vs. /ε/; Sebastián-Gallés and Soto-Faraco, 1999) but they nonetheless show poorer use of these distinctions in lexical decision and other higher level processing tasks (Pallier et al., 2001; Navarra et al., 2005; Sebastián-Gallés and Baus, 2005; Sebastian-Gallés et al., 2006; Díaz et al., 2008). Interestingly, the discrimination between Catalan sounds /e/ and /ε/ is enabled in Spanish-dominant Spanish-Catalan bilinguals who cannot otherwise distinguish these phonemes auditorily, when both the visual and the auditory speech information are available (Navarra and Soto-Faraco, 2007). This finding suggests that providing visual speech information can enhance discrimination of spoken L2 sounds.

Second language learners also show differences with regard to prosodic or supra-segmental language contrasts (e.g., Otake and Cutler, 1999). For instance, stress patterns on nonsense words are easily perceived by speakers of Spanish (a language in which stress can vary at the word level) but not speakers of French (a language in which stress is mostly invariant at the word level; Dupoux et al., 1997, 2008). Additionally, extensive training on some supra-segmentals (Mandarin tones) can lead to improvements in tone discrimination (Wang et al., 1999). However, in contrast to birth or very early bilinguals, adult L2 learners rarely achieve native-like performance.

Studies looking at the age of acquisition (AoA) of the second language suggest that the auditory phonemic system appears to start losing plasticity in early childhood. For example, among children who acquired a second language after age 7, auditory phonetic perception and production of accent-free speech are less precise than among children who acquired their second language before age 7 (e.g., Flege and Fletcher, 1992; Flege et al., 1995). Other studies indicate that even early bilinguals who learned their second language between birth and 6 years struggle on some phonological tasks in their second language (Pallier et al., 1997; Sebastián-Gallés and Soto-Faraco, 1999) and show, in general, poor sensitivity to phonetic distinctions from their non-dominant language when speech is presented acoustically (Navarra et al., 2005; Sebastián-Gallés and Baus, 2005). Early auditory language exposure thus seems important for achieving native-like phonological processing and accent-free fluency, though the age at which performance deteriorates can vary with the task.

Evidence concerning the importance of early experience for language acquisition also comes from studies of children and adults who, through adoption or immigration, had first language attrition to some degree while acquiring a second language. An influential series of studies tested adults who had been adopted from Korea between the ages of 3 and 9 into French homes and hence had little to no opportunity to speak or even hear Korean thereafter. These adults showed no savings from their early exposure to Korean, and were unable to recognize sentences or understand individual words in Korean (Pallier et al., 2003), or to discriminate the Korean 3-way distinction among plain, tense and aspirated voiceless Korean stops (not used in French; Ventureyra et al., 2004). Indeed, their performance on these speech contrasts was not significantly different from that of French speakers who had no exposure to Korean as children. In contrast, other studies have found lasting influences from the first language even years after it had attrited. For example, Korean adoptees to the U.S. were able to discriminate Korean words better than English listeners, particularly if they had some re-exposure to Korean (Oh et al., 2003). Moreover, studies following exposure to languages as diverse as Korean, Spanish, and Hindi—even just during the infancy period with subsequent loss of that first language—show a significant advantage in training studies or language learning classes for learning auditory phonetic contrasts from the attrited language (Tees and Werker, 1984; Au et al., 2002; Knightly et al., 2003; Oh et al., 2003, 2010; Hyltenstam et al., 2009). Thus, to the extent that retraining is seen as reactivation of old memory traces (e.g., Bjork and Bjork, 2006), one can say that exposure during the first few years of life can have a lasting effect on sensitivity to phonemic contrasts.

Despite all the research in speech perception, the vast majority of studies deal with auditorily presented materials. Much less is known about the development of visual speech perception capabilities. As previously mentioned, monolingual infants aged 4 and 6 months are able to discriminate their native language from an unfamiliar language just by watching silent talking faces, but no longer do so by 8 months unless they are growing up in a bilingual environment (Weikum et al., 2007; Sebastián-Gallés et al., 2012). Nonetheless, there is some latent sensitivity to visual information even among adults, but only if they know one of the languages. For example, Soto-Faraco et al. (2007) found that adult Spanish, Catalan, and Spanish-Catalan bilinguals were able to discriminate visual Spanish from visual Catalan significantly better than chance, whereas Italian and English speakers were not. Using two languages that were less similar, English and Spanish, Ronquest et al. (2010) reported similar results.

A question that these studies do not address is whether there is an influence of AoA for one of the test languages on visual language processing, in the same way that this variable plays an important role in auditory language perception. There is one suggestion in the literature of such an effect in a study of visual language discrimination of Finnish vs. Swedish where a trend was observed for better discrimination by participants' age of arrival in Sweden (Öhrström et al., 2009). The current study investigated precisely this question: Does age of acquisition of an L2 play a role in the ability to visually discriminate the L2 language from other languages? In order to investigate this issue, we tested adult participants from varied (non-French) language backgrounds who had acquired English at different ages (from birth to late childhood) on the visual French and English stimuli (used in previous work with infants, Weikum et al., 2007; Sebastián-Gallés et al., 2012). English and French differ both rhythmically and phonetically. Rhythmically, the two languages differ as English is a stress-timed language and French is a syllable-timed language (Pike, 1945; Abercrombie, 1967). Phonetically, segmental differences, such as more vowel lip-rounding and greater degree of lip protrusion in French, and the use of interdental articulations in English, exist between the two languages (Benoit and Le Goff, 1998).

On the basis of the literature reviewed above, showing age of acquisition effects on phonetic (segmental) and supra-segmental auditory speech perception, we hypothesized that visual language discrimination would also be influenced by the age at which the second language was learned. We therefore tested adults who had learned English at different ages. We divided the adults into three groups. The first group (Infant Exposure) was comprised of adults who had acquired English in infancy (by 2 years)—either as a single language or in a dual language-learning environment. Because an effect has been found for visual language discrimination between 6- and 8-months (Weikum et al., 2007), we were interested to determine whether this decline in visual language discrimination provides evidence for an optimal period in infancy that has life long consequences, or whether it shows a (re)organization process that has begun, but has not yet become permanent. However, adults are not accurate in reporting precisely when input from a second language began (especially if it was early in life), so we decided to use a broad range (0–2) to cover infancy. Thus, although a cut-off at 6 months of age would have provided an ideal comparison for the perceptual change found in the infant work, to be conservative we used a 2 year cut-off. The second group (Early Exposure) was comprised of adults who had acquired English after age 2 and before 6 years. Previous studies examining auditory speech perception and production have suggested that age 6 may be an important cut-off for phonological processing and accent-free speech (e.g., Flege and Fletcher, 1992; Flege et al., 1995) and studies have also shown that even early bilinguals may show differences on difficult phonological tasks (Pallier et al., 1997; Sebastián-Gallés and Soto-Faraco, 1999). Thus, this middle age group was comprised of Early, but not “crib” bilinguals. From a theoretical perspective, this group would include individuals who acquired the second language once the perceptual reorganization for the first language had already been established. The third group (Late Exposure) was comprised of adults who had acquired English after age 6 and before age 15. We compared these three groups on their ability to discriminate English visual speech from French visual speech (a non-native language for all the participants).

We predicted that the adults' ability to discriminate English from French based on visual information alone would depend on the age at which they learned English. To control for the possibility that short-term familiarity with a speaker could enhance language discrimination, we showed all participants videos of three different bilingual speakers and tested participants under two conditions. In the random condition, paired sentences from all three speakers were presented in random order. In the blocked condition the participants viewed all the sentence pairs from each of the three speakers in succession. If the blocked condition (where participants were able to see the same speaker over and over) conferred any short-term familiarity benefits, we would expect improved performance among the speakers in the blocked condition.

## Methods

### Participants

In accordance with the Behavioral Research Ethics Board at the University of British Columbia, all participants gave informed consent before participating. There were 120 adult participants (see Table 1 for details). Sixty participants had learned English as a first language (L1) before age 2. In this group, 40 participants had learned only English and 20 participants had learned English in conjunction with another language (Infancy multilinguals). An additional group of 60 had learned English as a second language (L2) after the age of 2 years. These L2 participants were further divided according to the age at which they started to learn English. Thirty participants had learned English as a second language in early childhood (age 2–6 years; Early multilinguals), and 30 participants had learned English learned as a second language in late childhood (age 6–15 years; Late multilinguals). Although the first language (L1) of the L2 participants was quite varied, the majority of the languages were either Cantonese or Mandarin (see Table 2 for participant language background information). None of the participants were fluent in French^1^.

**Table 1:** **Participant Data**.

	***N***	**Age English learned**	**Male/Female**	Mean age in years at test (*SD*)^*^
**L1**				
English only	40	0–2	21M/19F	25.3 (7.1)
Infant multilinguals	20	0–2	9M/11F	21.1 (3.1)
**L2**				
Early multilinguals	30	2–6	11M/19F	20.5 (2.1)
Late multilinguals	30	6–15	13M/17F	21.2 (4.2)

* *Age at test was only available for 109 participants*.

**Table 2:** **Multilingual participants' other language data**.

	**Infancy**	**Early**	**Late**	**Total**
Cantonese	8	21	9	38
Mandarin	1	3	9	13
Arabic	0	1	0	1
Danish	0	0	1	1
Farsi	1	0	0	1
Filipino	2	0	0	2
German	1	0	2	3
Hebrew	0	0	1	1
Indonesian	0	0	1	1
Japanese	1	0	0	1
Korean	0	1	2	3
Polish	0	0	1	1
Punjabi	3	2	1	6
Russian	1	2	3	6
Spanish	1	0	0	1
Tamil	1	0	0	1
Total	20	30	30	80

All subjects were highly proficient in English. All courses at the university they were attending were in English, and all who had English as a second language had passed the mandatory TOEFL requirement. In addition, we asked participants who had learned English as a second language, or simultaneously with another language from birth to rate themselves on their English proficiency. The first 11 participants rated their proficiency on a 7-point Likert scale where (1) represented native-like and (7) represented beginner. We switched to a more detailed questionnaire (Desrochers, 2003) for the remaining participants. This included 8 oral comprehension and 14 oral production questions. For each question, participants rated the difficulty of various speech activities on a 9-point Likert scale as very easy (1) to very difficult (9). The mean answer to these 22 questions was used as each participant's proficiency score. Proficiency in English was not available for 2 participants who had learned English simultaneously with another language.

### Stimuli

The faces of three balanced bilingual (French/English) speakers were recorded while they recited sentences in both English and French. The French and English sentences were taken from the French and English versions of the book “The Little Prince,” and were selected to overlap in content (same sentence translations) and to be roughly equivalent in length (see **Appendix** for examples). The sentences from each language were then individually digitized with the sound removed, to create 8–13 s silent video clips. There were no significant differences between sentence lengths for the English [average 37.24 (*SD* = 6.00) syllables] and the French [average 33.24 (*SD* = 5.88) syllables] video clips.

### Procedure

Participants were tested in a sound-attenuated room and sat at eye level with the monitor (17″) of a Pentium 4 PC. From a distance of ~75 cm, the participants watched 24 pairs of sentences, and each pair was played consecutively. For each pair of sentences, a white fixation point would first appear in the center of the black screen for 500 ms. Following this, a red frame with the speaker silently reciting one of the sentences would appear and was followed by a 1 s interval of black screen before the second sentence in the pair was played inside a green frame. Participants were asked to press the right mouse button (marked with an S) if they thought both clips were in the same language and the left mouse button (marked with as D) if they thought that they were from different languages. During the second sentence (green frame) participants had been instructed to respond as soon as they were sure of their judgment. If a response was not made during the second sentence, a white question mark appeared in the center of the black screen and was displayed until a response was made or 2000 ms elapsed. The language for each sentence clip was chosen pseudorandomly by the computer for each participant. The order and total number of sentences was set to be equiprobable, with each sentence appearing only once.

The two sentences in a given trial were spoken by the same person and were different in content. In the random condition, the clips used in a given trial were selected randomly from one of the three speakers. In the blocked condition, eight clip pairs from each individual speaker were presented consecutively before moving on to the eight pairs from the next speaker. This allowed for a test of potential improvement across exposure to each speaker. The order of the speakers was counterbalanced for each condition and the speaker order for the blocks was counterbalanced across participants.

## Results

**Figure 1:**
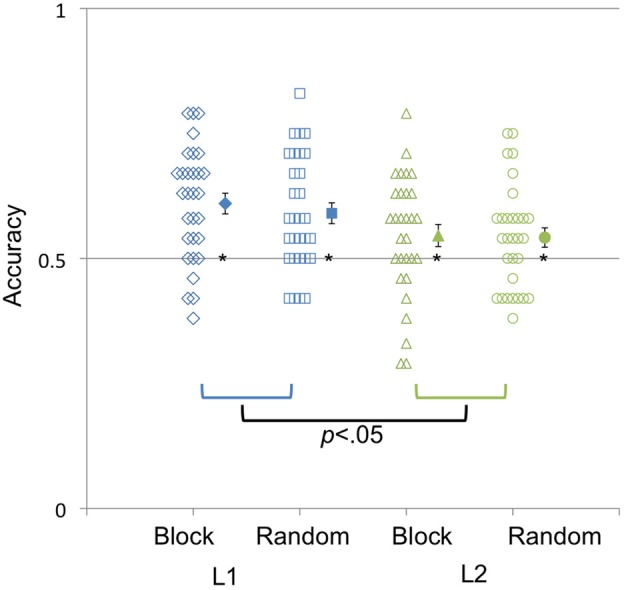
**Accuracy (percentage correct) in identifying whether silent video clips were from the same or different languages in both Random and Blocked speaker orders.** The *y*-axis represents mean accuracy; the *x*-axis represents whether the adults had learned English before age 2 (L1) or after the age of 2 years (L2). Filled-in symbols represent the group means. Error bars represent the standard error of the mean. ^*^p < 0.05.

Using group mean averages, a series of one-sample *t*-tests revealed that across all ages of acquisition, both the English L1 (English learned alone in infancy or simultaneously with another language) [*M* = 60%, *t*_(59)_ = 6.84, *p* < 0.001] and English L2 (Early and Late multilinguals) [*M* = 54%, *t*_(59)_ = 3.00, *p* < 0.05] discriminated the languages significantly better than chance, and did so in both the Random [*M* = 57%, *t*_(59)_ = 4.56, *p* < 0.001] and Blocked [*M* = 58%, *t*_(59)_ = 4.99, *p* < 0.001] speaker blocks. A univariate analysis of variance (ANOVA) including sex, language background (English as L1 or English as L2), and speaker order (blocked or random) yielded only a significant main effect for language background [*F*_(1, 119)_ = 8.08, *p* < 0.05; Figure 1]. Simple main effect analyses showed that the English L2 speakers performed significantly worse than the English L1 speakers [*F*_(1, 119)_ = 5.40, *p* < 0.05].

**Figure 2:**
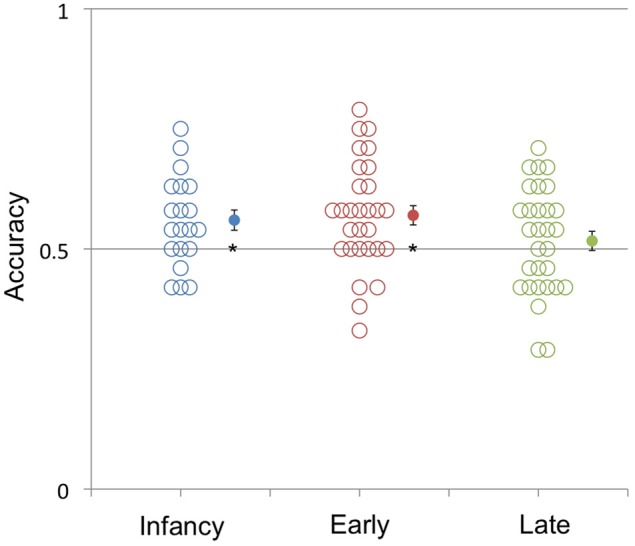
**Accuracy in identifying whether silent video clips were from the same or different languages of multilingual adults who had learned English: simultaneously with another language before age 2 (Infancy), between age 2 and 6 (Early), and after the age of 6 (Late).** The *y*-axis represents mean accuracy and the *x*-axis represents the age at which English was learned. Filled-in symbols represent the group means. Error bars represent the standard error of the mean. ^*^p < 0.05.

To probe whether age of acquisition of English had an effect on visual speech discrimination, we ran additional analyses. An ANOVA analyzing the effect of age of English acquisition (age 0–2, 2–6, 6–15) yielded a significant effect [*F*_(2, 117)_ = 5.55, *p* < 0.05]. Planned comparisons focusing on the multilingual participant groups revealed that the Infant and Early multilingual age groups did not perform significantly different from each other [*F*_(1, 48)_ = 0.24, *p* = 0.63], but did perform better than adults who acquired English in late childhood (6–15 years) [*F*_(1, 78)_ = 3.90, *p* = 0.05]. In fact, performance was significantly better than chance for multilingual learners who acquired English in *M* = 56%, *t*_(19)_ = 2.69, *p* < 0.02] and learners who acquired English in early childhood [*M* = 57%, *t*_(29)_ = 3.53, *p* < 0.02], but not for participants who acquired English in late childhood [*M* = 52%, *t*_(29)_ = 0.82, *p* = 0.417]. These results are graphically illustrated in Figure 2, which reveals as well that the vast majority of subjects in the infancy and early childhood groups, but not in the late English acquisition group, performed better than chance.

We performed several follow-up analyses with the multilingual groups in order to explore whether proficiency or number of years of experience, rather than age of acquisition (see Flege et al., 1997), could account for our findings. There was no significant correlation between discrimination performance and self-rated proficiency in English [*r*_(77)_ = −0.18, *p* = 0.12]^2^. Correlating discrimination performance with total years of experience with English [*r*_(70)_ = 0.09, *p* = 0.48]^3^, and exposure to French [*r*_(79)_ = 0.02, *p* = 0.84] also failed to reach significance. However, there were significant group differences between the means for proficiency scores, 1.16 (Infant multilinguals), 1.48 (Early multilinguals), and 1.95 (Late multilinguals), [*F*_(2, 74)_ = 5.92, *p* < 0.01] as well as the group means for years of experience, 20.1 (Infant multilinguals), 16.5 (Early multilinguals), and 12.4 (Late multilinguals), [*F*_(2, 65)_ = 40.14, *p* < 0.01].

To further probe the possibility that self-rated proficiency or years of experience with English may have contributed to our findings, we equated the Early and Late Multilingual groups by selecting subsets with equivalent proficiency scores or years of experience. We selected a subset of Late multilinguals who scored between 1 and 3 on the proficiency scale [with a mean score = 1.48(0.67) that was equivalent to the Early multilinguals = 1.53(0.60)]. The results from the full sample concerning the influence of AoA were replicated in the restricted Late multilingual sample as the late learning multilinguals again failed to perform significantly better than chance [*M* = 53.3%, *t*_(22)_ = 1.47, *p* = 0.16].

Similarly, we also tested the effect of AoA by selecting a subset of English L2 speakers who had an equivalent amount of experience in total number of years (12–19 years), and then within this group, compared the effects of early and late AoA. This resulted in 2 groups: 20 early bilinguals with a mean = 15.3(1.26) years of experience and 16 late bilinguals with a mean = 14.06(2.17) years of experience, wherein the mean years of exposure were not significantly different. The results from the full sample concerning the influence of AoA were replicated in this restricted sample: early bilinguals performed significantly better than chance [*M* = 56.0%, *t*_(19)_ = 2.79, *p* < 0.05] while the late learning bilinguals did not [*M* = 52.6%, *t*_(15)_ = 0.96, *p* = 0.35].

## Discussion

The age at which a language is learned (in this case, English) during childhood influences the ability to visually discriminate this language from others in adulthood. Interestingly, this effect of AoA could be examined separately from the influence of years of exposure or proficiency (self-rated). When tested on a visual language discrimination task, most participants who had learned English as a second language in late childhood (after 6 years) failed to discriminate English from French, whereas most participants who had learned English earlier, as infants (0–2 years old) or in early childhood (2–6 years old), succeeded. Allowing the participants to view the speakers in a blocked vs. random speaker order did not seem to have an influence on discrimination performance.

According to prior research, infants who are familiar with both languages (French and English since early infancy) retain the capacity to continue discriminating the languages visually at 8 months, while their monolingual counterparts fail (Weikum et al., 2007). This benefit arising from bilingual exposure appears to confer an advantage in adulthood too, as adults familiar with both test languages perform visual language discrimination significantly better than those familiar with only one of the test languages (Soto-Faraco et al., 2007). Based on the infant research, one might argue that the successful discrimination of French and English by monolingual English infants at 4 and 6 months, followed by a decline at 8 months, predicts that monolingual English adults should also fail to discriminate English and French (Weikum et al., 2007). However, the present findings (see also Soto-Faraco et al., 2007 for converging results) show that monolingual participants do indeed successfully discriminate their native language from an unfamiliar language. One reason adults succeed and older infants do not, may be that adults are able to use a wider and more sophisticated range of strategies to resolve the task. However, if it was only strategy on the part of the monolingual adults that leads to their success in language discrimination, then the failure of our English L2 late learning adults to tell apart French from English is surprising. Instead, our results suggest that exposure to one of the languages any time before age 6 allows for continued discrimination in adulthood.

Sensitive periods have been previously identified for phonemic segment discrimination in auditory spoken languages (for a review see Werker and Tees, 2005) and for acquisition of syntax in signed languages (Newport, 1990). The results from this study further support these findings by showing that sensitive periods also exist for language discrimination based on visual speech cues alone. Although it was not the intention of this study to address what these cues may be (see Soto-Faraco et al., 2007; Ronquest et al., 2010; Navarra et al., submitted), for work investigating the role of visual phonetic and rhythmical cues), our results suggest that some visual language cues are subject to sensitive periods. On the other hand, some of the subjects in the late acquisition group did succeed at discriminating visual French from visual English. Thus, either some cues are subject to sensitive period effects and others are not, and the subjects differentially attended to these cues, or there are individual differences between the subjects such that some retain greater openness to non-native information than do others. Understanding this within group variability more deeply will be an important focus for future research. It will provide insight into the speech perception limitations faced by both first and second language learners, and provide guidance for improvement.

### Conflict of interest statement

The authors declare that the research was conducted in the absence of any commercial or financial relationships that could be construed as a potential conflict of interest.

## Footnotes

^1^One of the subjects in the Late multilingual group whose first language was Mandarin subsequently became proficient in both French and English, but no longer uses French.

^2^The data for this analysis were only available for 78 of the 80 participants and 1 participant's data was removed as their proficiency score was more than 3 *SD* from the mean.

^3^The data for this analysis were only available for 71 of the 80 participants.

## Appendix

### Sentence examples from the book, le petit prince/the little prince by antoine de saint-exupery

#### Sentence 1

English version- The little prince had watched very closely over this small sprout which was not like any other small sprout on this planet.

French version- Le petit prince avait surveillé de très près cette brindille qui ne ressemblait pas aux autres brindilles.

#### Sentence 2

English version- If the two billion inhabitants who people the surface were all to stand upright, all humanity could be piled up on a small Pacific islet.

French version- Si les deux milliards d'habitants qui peuplent la terre se tenaient debout et un peu serrés, on pourrait entasser l'humanité sur le moindre petit îlot du Pacifique.
